# 

**Published:** 1897-07-03

**Authors:** 


### EARLY EDITION.

**Notices of Births, Deaths, and Marriages** are inserted in this column at a charge of 2s. 6d. for an announcement not exceeding four lines.

### NOTICE TO CORRESPONDENTS.

All MS., Letters, Books for Review, and other matters intended for the Editor should be addressed The Editor, "The Hospital," The Hospital Building, 28 & 29, Southampton Street, Strand, W.C.

The Editor cannot undertake to return rejected MS., even when accompanied by stamped directed envelope.

**Notice to Advertisers and Subscribers.**

All Advertisements, Orders for Copies of The Hospital, and Business Communications should be addressed to **THE MANAGER, (Not to The Editor).** The Hospital, The Hospital Building, 28 & 29, Southampton Street, Strand, London, W.C.

The Cost of Subscription, post free, is as follows:—Per week, 2½d.; per quarter, 2s. 8d.; per half-year, 5s. 3d.; per annum, 10s. 6d. Foreign:—Per annum, 15s. 2d.; payable, in all cases, in advance.

**NOTICE.—Vol. XXI. of "The Hospital" (October, 1896, to March, 1897), in handsome cloth binding, is on sale at the Office of this Journal, price 7s. 6d. Cases for binding the Numbers contained in this Volume, 1s. 6d. Index to Vol. XXI., 2½d., post free.**

**The Monthly Part for July is now ready. Price 6d., by post 8d.**

### BOOKS RECEIVED.

R. Morgan.

"The British Weather Chart, 1897." By B. G. Jenkins, F.R.A.S.

Sampson Low, Marston, and Co.

"Twentieth Century Practice of Modern Medical Science. Ninth volume.

Bailliere, Tindall, and Cox.

"The Analysis of Food and Drugs." Part 1, "Milk and Milk Products." By T. H. Pearman and C. G. Moor, M.A.

**Periodicals and Pamphlets Received.**—London, Literary Digest, South African Medical Journal, Medical Record, Cassell's Family Magazine, Nursing Notes, Our City, Knowledge. Westminster Review, New Age, Vegetarian, Toilers of the Deep, Annual Report Zenana, Medical Mission, Woman's Signal, Electrical Review, Trained Nurse, Mothers and Daughters, Industries and Iron, London Visitor, Excelsior, The Guardian, Charity Organisation Review, Guy's Hospital Gazette, Medical Record New York, Leisure Hour, Boy's Sunday Monthly, Sunday Hours, Scalpel, Boy's Own Paper, Sunday at Home, Friendly Greetings.

### AROUND THE HOSPITALS.

### SCRAPS AND GLEANINGS.

THE foundation-stone of the new hospital at Newport, Mon., is to be laid on August 2nd.

THE new premises of the Voluntary Lock Hospital at Bristol were opened on June 10th.

THE Royal Berks Hospital has been given £7,000 by Messrs. Huntley and Palmer to provide additional beds.

WE are pleased to note that in 1896 the subscriptions to the Llanelly Hospital were increased by about £350.

THE £100,000 required for the building of the Royal Victoria Hospital at Belfast has nearly all been collected.

THE Mayor's Fund to extinguish the debt on the Southport Infirmary has been closed, the full amount asked for having been subscribed.

THE average number of cases attended to at the Lawn Hospital for Mental and Nervous Diseases, Lincoln, during the past year, was 78.

THE report of the managers of the Leicester and Leicestershire Provident Dispensary shows the total on the books of membership to be 42,148.

THE proceeds of the annual sports of the Royal Irish Constabulary were divided between the Victoria and the Mater Infirmorum Hospitals at Dublin.

AN excellent suggestion has been made at Margate, viz., that instead of enlarging the old cottage hospital, as has been proposed, a new building should be erected on a fresh site.

A DONATION of £300 has been received by the committee of Paddington Green Children's Hospital towards the expenses of the convalescent home at "The Laurels," Wealdstone, Harrow.

CLOSE upon 3,750 out and 250 in patients were admitted to the Newcastle Eye Infirmary last year. There was a deficit on the year's working of £313, and the debit balance at the end of the year was £293.

THE daily average number of in-patients treated in 1896 at the Pembrokeshire and Haverfordwest Infirmary was 18, as compared with 16 in 1895. The debit balance of £83 for 1895 had been increased to £134 in 1896.

THE total Hospital Saturday collection at Stafford, on behalf of the Staffordshire General Infirmary this year, was £258, against £259 collected last year. The net sum (after deduction of expenses) handed over to the infirmary was £226, compared with £211 last year.

THERE have been 534 in-patients treated at the Gravesend Hospital during the year ended March 31st, 1897, of whom 261 were men, 129 women, and 144 were children. The out-patients numbered 5,585, and the district nurse made 3,086 visits to the poor in their own homes.

THE Worshipful Company of Salters and the Worshipful Company of Merchant Taylors have each given a donation of £10 10s. to the funds of the After-Care Association for Poor Persons Discharged Recovered from Asylums for the Insane, of which association the Earl of Meath is president.

THE President of the Devon and Exeter Hospital pointed out at the annual meeting the other day that out of the £1,811 received in subscriptions in 1896 £1,107 came from the county of Devon, and £704 from the town of Exeter. Of the patients treated 2,172 came from the county and 3,357 from Exeter. Out of the 50,000 people in Exeter, Heavitree, and St. Thomas only 186 subscribed to the hospital.

THE scheme contemplated by Mr. Quarrier for providing hospitals for the treatment of consumption at Bridge of Weir is to erect six hospitals containing in all about 200 beds, which, with executive buildings, lodge, &c., will cost about £60,000. Towards this sum about £20,000 has been received, which has been expended on the first hospital (opened in September, 1896), executive buildings, baths, lodge, &c. The cost of each hospital, providing for from 35 to 40 patients, is estimated at £8,000.

MR. KEITH D. YOUNG has been consulted by the Board of Management of the Huntingdon County Hospital as to the improvements which should be made in the institution in order to bring it more into line with present day requirements. His recommendations include the erection of a new operating room, the re-flooring of all the wards with teak, and the rearrangement of the drainage. Besides this the Board are anxious to provide an isolation ward and a new mortuary. In order to carry out the necessary alterations, the sum of £2,900 is required.

IN his annua report on the health of Norwich in 1896, Dr. Cooper Pattin, Medical Officer of Health, comments strongly on the inadequacy of the fever hospital. "We ought," he says, "to have beds provided in the proportion of 1 per 1,000 of the population; as a matter of fact, we have only one bed to every 2,250 of the people. The lack of hospital accommodation was responsible for quite 40 per cent. of the cases of scarlet fever which occurred during the latter part of the year. Our being unable to remove the first case from a dwelling constantly led to the occurrence of more cases in the family or immediate neighbourhood."

ONE of the pleasantest features of our modern commercial life is the spirit of friendly co operation which exists between employer and employed, and the firm of Messrs. E. Cook and Co., the soap manufacturers, of Bow, are a striking example of the pleasant results of such action. On the occasion of a dinner given by them to their staff, the bonuses for the past year were distributed, and in a few days' time bonuses are to be distributed to all the workmen who have been in the employ of this firm during 1896. Messrs. Liebig, too, take a kindly interest in the welfare of their employés. These number over 2,000, and the company have provided all kinds of recreation rooms, besides baths for the workmen.

THE history of hospital work in Coventry is interesting, In 1831 two dispensaries—the Self-Supporting Dispensary (now called the Provident Dispensary) and the General Dispensary—were formed. The latter, in 1837, grew into a small general hospital, which was situated at the top of Little Park Street. Greater hospital accommodation becoming necessary, the present hospital on Stoney Stanton Road was erected in 1864. In 1873 a movement in favour of establishing a children's hospital was started, which subsequently resulted in the furnishing of a wing of the Coventry and Warwickshire Hospital as children's wards. The City or Fever Hospital was erected by the Corporation in 1884, and a small-pox hospital is shortly to be provided by the same body.

### The Student of Medicine.

**APPOINTMENTS.**

J. Anderson, M.B., C.M.Edin., appointed Parochial Medical Officer for the Eastern Division of the Parish of Logierait and Parochial Medical Officer. J. G. Bailey, C.M.Edin., appointed Junior House Surgeon to the Huddersfield Infirmary. Dr. Barker, appointed Medical Officer for the Haxey District of the Gainsborough Union. R. A. Bayliss, M.R.C.S., L.R.C.P.Lond., appointed Resident Medical Officer to the Royal Mineral Water Hospital, Bath. Dr. Blackmore, appointed Temporary Assistant Medical Officer to the Workhouse of the Nottingham Union. J. A. Cameron, M.B.Glasg., appointed Medical Officer for Glenorchy and Inishail Districts of the Argyll County Council. T. Carwardine, M.B., M.S.Lond., F.R.C.S.Eng., appointed Assistant Surgeon to the Bristol Royal Infirmary. A. H. Copeman, B.A.Camb., M.R.C.S.Eng., appointed Medical Officer for the Littleport District of the Ely Union. R. F. Cox, L.R.C.P.Lond., M.R.C.S.Eng., appointed Medical Officer for the Knighton District of the Knighton Union. A. Dingwall, M.B.Aberd., appointed Medical Officer of Health to the Presteign Urban District Council. A. R. Douglas, L.R.C.P., L.R.C.S., appointed Deputy Medical Officer at H.M. Prison, Portland. E. W. Du Buisson, M.R.C.S., L.R.C.P., appointed Honorary Surgeon to the Hereford General Infirmary. S. Edgerley, M.A., M.B., appointed Second Assistant Medical Officer to the West Riding Asylum, Menston. H. D'Arcy Ellis, L.C.R.P.Edin., M.R.C.S., appointed Medical Officer of Health to the Kingswinford Rural District. J. Farrar, M.D.St.And., L.R.C.P.Edin., appointed Medical Officer for the Workhouse and the Gainsborough District of the Gainsborough Union. P. Flemming, F.R.C.S., M.D., B.S.Lond., appointed Assistant Ophthalmic Surgeon to University College Hospital. A. D. Fripp, M.S., appointed Assistant Surgeon to Guy's Hospital. H. Harris, M.D., appointed Medical Officer for the Ninth District of the Bedminster Union. F. Harwood, appointed Medical Officer to the Brentford Union Infirmary. J. G. Havelock, M.D.Edin., appointed Physician Superintendent to the Royal Asylum, Sunnyside, Montrose. R. D. Helm, M.D., appointed Physician to the Cumberland Infirmary. J. E. Howden, M.D., appointed Consulting Physician to the Royal Asylum, Sunnyside, Montrose. H. W. Jackson, M.B.Lond., L.R.C.P., M.R.C.S., appointed House Physician to the Royal Berkshire Hospital, Reading. H. Keighley, M.R.C.S.Eng., L.R.C.P.Lond., appointed Medical Officer for the Batley District of the Dewsbury Union. C. Latter, M.D., B.C., B.A.Cantab., appointed Honorary Medical Officer to the Victoria Hospital, Folkestone. D. R. McArthur, M.D.Edin., reappointed Medical Officer for the East Ward District of the Luton Union. J. M'Donnell, L.R.C.P.I., L.R.C.S.-Edin., appointed Medical Officer to the Roscommon Dispensary District. A. MacNaughton, M.D.Glasg., appointed Medical Officer for Kilchrenan and Dalvaich District of the Argyll County Council. E. W. Marshall, L.R.C.P., M.R.C.S., appointed Medical Officer for the Sixth District of the Croydon Union. J. R. Marshall, M.B., C.M.Glasg., appointed Medical Officer of Health for Carriden. F. H. Marson, M.B., M.D., B.S.Durh., L.R.C.P.Lond., F.R.C.S.Eng., appointed Honorary Surgeon to the Staffordshire General Infirmary. G. Martin, L.R.C.S.I., L.R.C.P.I., appointed Visiting Medical Officer to the Cumberland and Westmorland Convalescent Home. E. F. Maynard, M.D.Edin., appointed Assistant Physician to the Sussex County Hospital. G. Michelmore, M.R.C.S.Eng., appointed Medical Officer for the Workhouse of the Tiverton Union. H. F. Mole, F.R.C.S.Eng., appointed House Surgeon and Senior Resident Officer to the Bristol Royal Infirmary. P. F. Monaghan, L.R.C.P., L.R.C.S.I., appointed Medical Officer for the Kiltormier Dispensary District. W. H. Moore, M.R.C.S.Eng., L.R.C.P.Lond., appointed Medical Officer for the Workhouse of the Kidderminster Union. C. P. Moreton, M.R.C.S.Eng., L.S.A., appointed Medical Officer of Health to the Forden Rural District. G. Nicholl, M.B.Glasg., appointed Medical Officer of Health to the Clyne Parish Council. W. Pasteur, M.D.Lond., appointed Consulting Physician to the North-Eastern Hospital for Children, Hackney Road.

